# Mechanisms of Disease: Host-Pathogen Interactions between *Burkholderia Species* and Lung Epithelial Cells

**DOI:** 10.3389/fcimb.2015.00080

**Published:** 2015-11-18

**Authors:** Jonathan David, Rachel E. Bell, Graeme C. Clark

**Affiliations:** ^1^Microbiology, Biomedical Sciences, Defence Science and Technology LaboratorySalisbury, UK; ^2^Division of Immunology, Infection and Inflammatory Disease, Centre for Molecular and Cellular Biology of Inflammation, King's College LondonLondon, UK

**Keywords:** *Burkholderia*, epithelium, epithelial, lung, host-pathogen interaction

## Abstract

Members of the *Burkholderia* species can cause a range of severe, often fatal, respiratory diseases. A variety of *in vitro* models of infection have been developed in an attempt to elucidate the mechanism by which *Burkholderia* spp. gain entry to and interact with the body. The majority of studies have tended to focus on the interaction of bacteria with phagocytic cells with a paucity of information available with regard to the lung epithelium. However, the lung epithelium is becoming more widely recognized as an important player in innate immunity and the early response to infections. Here we review the complex relationship between *Burkholderia* species and epithelial cells with an emphasis on the most pathogenic species, *Burkholderia pseudomallei* and *Burkholderia mallei*. The current gaps in knowledge in our understanding are highlighted along with the epithelial host-pathogen interactions that offer potential opportunities for therapeutic intervention.

## Introduction

The lung epithelium is increasingly being acknowledged as having an important and complex role in protecting the body from infection. Aside from the obvious physical barrier properties the epithelium offers to the underlying endothelium and circulatory system, this region of the lung is also thought to have immunomodulatory roles which help during both the early phases of infection and aid in the resolution of the host response (Tam et al., 2011). Having a clear understanding of how microorganisms interact with the different regions within the lung will be essential in order to design new or novel medical treatments for combating infection. In order to achieve this aim a plethora of *in vitro* cell models have been developed in order to study infectious diseases.

## Modeling the respiratory tract

Epithelial cells change in their morphology and function throughout the respiratory tract. As a consequence, a range of different *in vitro* systems have been developed for oral, nasal, laryngeal, mucoepidermoid, bronchiolar, and alveolar cells in order to establish the architecture and key roles of each region within the tract. However, these models also provide a vital means of establishing how infectious organisms can interact with this first line of defense in the body. As bacteria travel the length of the respiratory tract they will come into contact with these epithelial cells but also other cell types including; lymphocytes and macrophages in the sub-epithelia region, mucosa-associated lymphoid tissue (MALT), bronchi-associated lymphoid tissue (BALT), basal cells, goblet cells, Clara cells, and alveolar macrophages. As the infection continues to progress, further immune cells (e.g., lymphocytes, eosinophils, and neutrophils) migrate into the lung. The lung environment is a complex construct of structural, secretory, and immune cells with all of these cells having the potential to interact with bacteria. This review focuses solely on the interactions of the lung epithelium with the Genera *Burkholderia*.

Epithelial cells are ubiquitous in the body and line many mucosal and tissue surfaces, including the respiratory tract. The epithelial layer is a vital barrier for protection against infection and cells are closely associated by tight junctions and other adherins (Parker and Prince, 2011). In the case of the respiratory tract, the epithelial layer is the primary defense against inhaled pathogens and is important to study the process of lung infections (Burns et al., 1996). Various *in vitro* models of infection have been developed to date to study the interactions between the host cells and *Burkholderia* spp. (Table 1; Eagle et al., 1956; Moorhead, 1965; Stoner et al., 1975; Lieber et al., 1976; Fogh et al., 1977; Carney et al., 1985; Chen, 1988; Reddel et al., 1988; Zeitlin et al., 1991; Cozens et al., 1994). *Burkholderia pseudomallei* in particular has a broad tropism for epithelial cells. The organism can adhere to a range of human epithelial cell lines *in vitro* including those derived from alveolar, bronchial, laryngeal, oral, conjunctiva, and cervical locations (Brown et al., 2002; Essex-Lopresti et al., 2005). As well as acting as an important physical barrier from infection, epithelial cells can also produce a range of products that can either directly or indirectly affect bacterial colonization and survival within in the lung, through the activation of arms of the innate response. These include antimicrobial products that act directly upon the invading organism and/or through the release of various cytokines in order to instigate an immune response leading to the recruitment of circulating monocytes required for the clearance of infection (Parker and Prince, 2011; Vareille et al., 2011). These direct and indirect responses will now be considered in more detail in the context of infections with *Burkholderia* spp. Whilst general interactions such as adherence, invasion and intracellular replication of the *Burkholderia* spp. have been consistently seen in a variety of cell types; it is also important to acknowledge cell specificity. Table 2 summarizes the research to date in this context.

**Table 1:** **Lung epithelial cell models used for studying *Burkholderia* infection**.

**Cell type**	**Species**	**Lung location**	**Derivation**
A549	Human	Alveolar	A type 2-like pneumocyte derived from adenocarcinoma (Lieber et al., 1976)
LA-4	Mouse	Alveolar	A type 2-like pneumocyte derived from adenocarcinoma (Stoner et al., 1975)
16HBE	Human	Bronchiolar	SV40 transformed bronchial epithelium (Cozens et al., 1994)
Calu-3	Human	Bronchiolar	Derived from a bronchial epithelial adenocarcinoma (Fogh et al., 1977)
BEAS-2B	Human	Bronchiolar	SV40/adenovirus 12 transformed bronchial epithelium (Reddel et al., 1988)
CFBE	Human	Bronchiolar	SV40/adenovirus 12 transformed cystic fibrosis bronchial epithelial cell line (Zeitlin et al., 1991)
NCI-H292	Human	Mucoepidermoid	Derived from a cervical node metastasis of a pulmonary mucoepidermoid carcinoma. These cells contain numerous small mucin-containing granules (Carney et al., 1985)
HEp-2	Human	Laryngeal	Originally thought to be from a laryngeal carcinoma it is now known to be established via HeLa cell contamination^*^ (Chen, 1988)
RPMI-2650	Human	Nasal	Derived from a malignant tumor of the nasal septum (Moorhead, 1965)
KB	Human	Oral	Originally thought to be from a carcinoma of the mouth it is now known to be established via HeLa cell contamination^*^ (Eagle et al., 1956)
Primary	Any	Any	Derived and cultured directly from tissue. Primary cells initially retain phenotypic characteristics of the donor tissue but do differentiate post isolation leading to variation in cell phenotype

*Numerous models of infection have been used to study the interaction of Burkholderia spp. with the epithelium. The location and derivation of these cell lines are shown*.

* *Numerous cell types have now been confirmed to be contaminated with HeLa cells (cervical cancer). After original isolation the HeLa cells out compete the originally derived cell lines and dominate the cultures*.

**Table 2:** ***Burkholderia* infection studies performed in lung epithelial cell types**.

**Biological process**	**Bacteria**	**Cell type**	**References**
Adherence	*B. pseudomallei*	A549	Brown et al., 2002; Kespichayawattana et al., 2004; Essex-Lopresti et al., 2005
		BEAS-2B	Essex-Lopresti et al., 2005
		RPMI-2650	
		NCI-H292	Brown et al., 2002
		HEp-2	
		KB	
Invasion	*B. pseudomallei*	A549	Jones et al., 1997; Tomich et al., 2002; Kespichayawattana et al., 2004; Chuaygud et al., 2008; Phewkliang et al., 2010
	*B. cepacia*	A549	Burns et al., 1996; Duff et al., 2006
		16HBE	Duff et al., 2006
		Calu-3	
		1^y^ Human	Schwab et al., 2002
	*B. cenocepacia*	16HBE	Mullen et al., 2007
		CFBE	
		1^y^ Human	Taylor et al., 2010
	*B. multivorans*	16HBE	Mullen et al., 2007
		CFBE	
		1^y^ Human	Schwab et al., 2002
Intracellular survival	*B. cepacia*	A549	Tipper et al., 1998
Intracellular replication	*B. pseudomallei*	A549	Chuaygud et al., 2008; Phewkliang et al., 2010
	*B. cepacia*	A549	Duff et al., 2006
		16HBE	
		Calu-3	
	*B. cenocepacia*	Immortalized CF epithelium	Sajjan et al., 2006
Bacterial movement	*B. cenocepacia*	Immortalized CF epithelium	Sajjan et al., 2006
Host response	*B. pseudomallei*	A549	Utaisincharoen et al., 2005; Wongprompitak et al., 2009
		LA-4	Bast et al., 2014
		1^y^ Murine	
	*B. mallei*	*in vivo*	Goodyear et al., 2010
	*B. thailandensis*	A549	Wongprompitak et al., 2009
	*B. cepacia*	A549	Palfreyman et al., 1997; Fink et al., 2003; Reddi et al., 2003; Mariappan et al., 2013
	*B. cenocepacia*	A549	Kaza et al., 2011
		16HBE	Kim et al., 2005; Kaza et al., 2011; Wright et al., 2011; Gillette et al., 2013
		Calu-3	Kaza et al., 2011; Gillette et al., 2013
		BEAS-2B	Gillette et al., 2013
		CFBE	Wright et al., 2011
	*B. multivorans*	A549	Kaza et al., 2011
		16HBE	
		Calu-3	
		CFBE	

## The pathogenic *Burkholderia* species

*Burkholderia* is a genus of Gram-negative Proteobacteria containing approximately 30 species. These species are associated with a range of diseases of varying severity in animals, plants and humans; often utilizing the lungs as the primary route of entry into the body. Of particular interest are *B. pseudomallei* and *B. mallei*, due to the severity of the diseases that they cause; melioidosis and glanders, respectively (Gilad et al., 2007). Both melioidosis and glanders can present in a range of forms and often with non-specific symptoms making early diagnosis extremely challenging (Limmathurotsakul and Peacock, 2011; Van Zandt et al., 2013). This represents a significant issue to clinicians given that, if left untreated, both diseases are associated with septic shock and high mortality rates; especially when contracted via the respiratory route. In addition, latent (chronic) infection also represents a significant issue in a clinical context by appearing to be able to reside asymptomatically within the body for years following an initial exposure. The longest recorded human incubation period is 62 years before clinical symptoms appeared with the bacteria remaining “dormant” during this timeframe (Ngauy et al., 2005). Disease relapse in treated patients can also recur years afterwards if the infection is not completely cleared with anti-microbial therapy (Limmathurotsakul and Peacock, 2011). The combined characteristics of acute and chronic infection make *B. pseudomallei* and *B. mallei* of particular concern from both a biodefence and public health perspective. Currently, medical therapeutic options are limited. No licensed vaccines are currently available for either melioidosis or glanders and due to natural resistance mechanisms held by the bacteria, treatment is restricted to a limited range of antibiotics. Even when treated with antibiotics mortality rates can be as high as 40% for cases of glanders (Van Zandt et al., 2013). With no licensed vaccines available antibiotic treatment remains the only option and is regularly required for many months to clear infection (Van Zandt et al., 2013). Medical guidelines currently states ceftazidime, meropenem, or imipenem with cilastatin should be used for intravenous treatment, followed by oral treatment with doxycycline and co-trimoxazole (H.C.f. Infections, 2008). Despite these prolonged antibiotic regimens, low levels of antibiotic resistance in clinical *B. pseudomallei* and *B. mallei* isolates have been observed (Heine et al., 2001; Wuthiekanun et al., 2011). However, resistance has been reported *in vitro* for *B. mallei* (Van Zandt et al., 2013), and in the clinical setting for less virulent *Burkholderia* spp. (Moore et al., 2001). For the successful identification of alternative treatments it is critical that the dynamic interplay between the bacteria and the host is understood. The interactions between bacteria and immune cells has previously been reviewed (Wiersinga and van der Poll, 2009; Silva and Dow, 2013) but the specific role of the lung epithelium during an infection with a *Burkholderia* sp. is an emerging field.

*B. pseudomallei* and *B. mallei* are highly pathogenic and therefore require Biosafety Level III containment for the safe handling and manipulation of the organism. *Burkholderia thailandensis* is less virulent than *B. pseudomallei* and is commonly used for modeling disease progression as it can be handled at lower levels of containment. Despite sharing some of these virulence mechanisms, *B. thailandensis* has a reduced virulence of 10^5^-fold in comparison to clinical *B. pseudomallei* strains (Brett et al., 1998). *B. thailandensis* has a genome with over 95% 16S rRNA homology with *B. pseudomallei* (Brett et al., 1998). The genome also contains various homologs of *B. pseudomallei* virulence factors including components of the type III secretion systems (T3SS; Brett et al., 1998; Haraga et al., 2008). The two commonly used strains of *B. thailandensis* utilized in laboratory research are, E264 and E555. *B. thailandensis* E555 has greater genetic homology to *B. pseudomallei* and also, unlike E264, expresses a similar capsule (Scott et al., 2013). *B. thailandensis* E264 does however possess a lipopolysaccharide (LPS) with a similar carbohydrate structure to that of *B. pseudomallei* (Ngugi et al., 2010). The choice of *B. thailandensis* strain should therefore be driven by research aims in light of these genetic and phenotypic differences. Most other *Burkholderia* spp. are saprophytic organisms that are generally associated with soil or plant material however some can cause infection as opportunistic pathogens affecting immunosuppressed individuals or causing secondary infection associated with an underlying disease condition, such as cystic fibrosis (CF) (Coenye and Vandamme, 2003). These species include *B. cepacia, B. cenocepacia*, and *B. multivorans*, which form the *Burkholderia cepacia* complex (Bcc). *B. cepacia* and *B. pseudomallei* are very closely related and have previously been misidentified by some commercial diagnostic techniques (Kiratisin et al., 2007; Zong et al., 2012). Although the differences between the Bcc and the highly virulent *B. pseudomallei* and *B. mallei* are still not fully characterized, there are similarities in their life cycles. This potentially allows findings relating to the mechanism of infections used by Bcc to be correlated to those generated by more virulent bacteria strains and hence provides an insight to these diseases (Lipuma, 2005). Notably, the ability to use less virulent infection models has enabled advances in the understanding of how the *Burkholderia* species interact with the epithelium.

### *Burkholderia* species: An introduction to virulence factors

**Figure 1:**
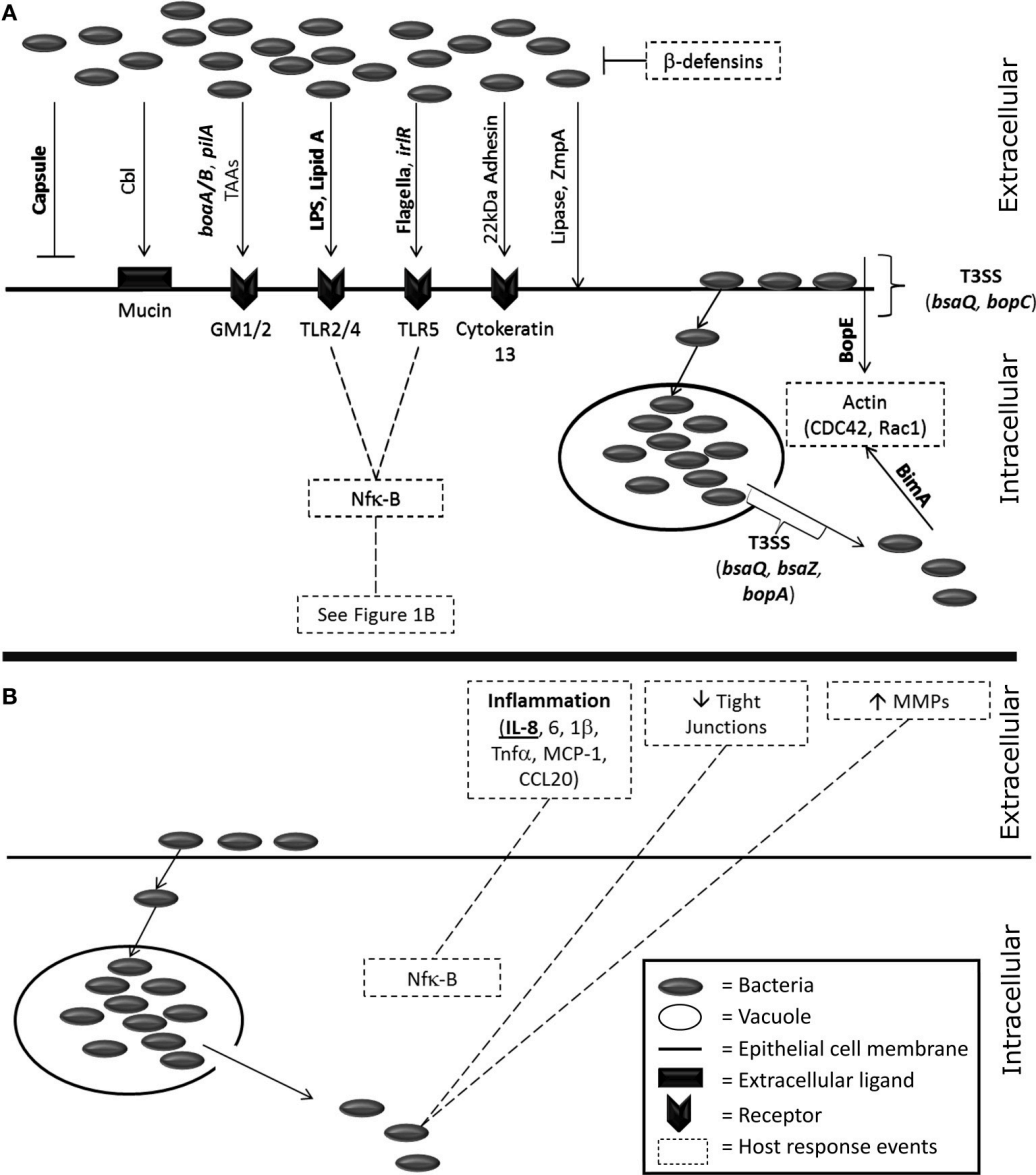
**A visualization of the known host-pathogen interactions of *Burkholderia* spp. with host epithelial cells**. **(A)** Bacterial factors known to interact with host epithelial cells. Items in bold are specific for *Burkholderia pseudomallei* and/or *mallei*. Invasion of bacteria is driven by several bacterial factors; capsule (Phewkliang et al., 2010), cable pili (Sajjan and Forstner, 1992, 1993), *pilA* (Essex-Lopresti et al., 2005), adhesins [*boaA/B* (Balder et al., 2010; Lu et al., 2012) and other auto-transporter adhesins (Mil-Homens and Fialho, 2012; Lafontaine et al., 2014)], LPS (Dziarski and Gupta, 2000), Lipid A (Dziarski and Gupta, 2000), flagella (Tomich et al., 2002; Chuaygud et al., 2008; Allwood et al., 2011), *irl* locus (Jones et al., 1997), a 22kDa adhesion (Sajjan and Forstner, 1993), lipase (Mullen et al., 2007), and the metalloprotease ZmpA (Gingues et al., 2005). Receptor binding events on epithelial cells occur via mucin (Sajjan and Forstner, 1992), the asialogangliosides GM1/2 (Gori et al., 1999), toll-like receptors (West et al., 2009, 2013), and cytokeratin 13 (Sajjan et al., 2002). Bacterial escape from vacuoles is driven by the T3SS (Pilatz et al., 2006; Gong et al., 2011) and once the bacteria are cytosolic BimA affects host actin polymerization (Stevens et al., 2005; Sitthidet et al., 2011). Direct entry into epithelial cells has also been linked to the T3SS and the effector protein BopE which also affects host actin (Rudolph et al., 1999; Stevens et al., 2003; Muangsombut et al., 2008; Muangman et al., 2011). **(B)** The host response to *Burkholderia* infection from epithelial cells. Inflammation is driven by Nfk-B induction (Dziarski and Gupta, 2000) of IL-8 (Palfreyman et al., 1997; Fink et al., 2003; Utaisincharoen et al., 2005; Sim et al., 2009; Lu et al., 2012), IL-6 (Sim et al., 2009; Lu et al., 2012) and IL-1β (Sim et al., 2009; Lu et al., 2012; Gillette et al., 2013), TNF-α, MCP-1 and CCL20 (Sim et al., 2009; Lu et al., 2012). Tight junctions are disrupted (Kim et al., 2005; Duff et al., 2006; Ferreira et al., 2015) and extracellular matrix components degraded by matrix metalloproteases (Wright et al., 2011).

Many virulence factors have been identified within pathogenic *Burkholderia* spp. and several of these have been shown to interact with the epithelium (Figure 1A). These interactions may offer the potential for therapeutic intervention. The best characterized example is the capsular polysaccharide which has been intensively studied for its role in disease progression and, as a consequence, has also been the focus for a number of therapeutic approaches (Reckseidler-Zenteno et al., 2009; Patel et al., 2011; Wang et al., 2011; Cuccui et al., 2012). The capsular polysaccharide aids in immune avoidance, specifically phagocytosis, and possesses homologous regions with the capsule from other respiratory pathogens including *Haemophilus influenza* and *Neisseria meningitidis* (Reckseidler et al., 2001). However, for alveolar epithelial cells (A549s) an acapsular mutant exhibited enhanced cellular internalization (Phewkliang et al., 2010). This suggests that, in this cell type at least, the capsule does not play a role in adhesion or invasion of *B. pseudomallei*. The secretion systems have also been identified as key virulence factors for *B. pseudomallei*. The genome contains six type VI secretion systems (T6SS) with cluster 1 identified as particularly important for the intracellular lifecycle of the organism (Burtnick et al., 2011). Additionally, *B. pseudomallei* contains three T3SS but only one has been recognized as important for virulence in humans with the other two found to have a role in plant models of infection (Haraga et al., 2008; Lee et al., 2010; D'Cruze et al., 2011). *Burkholderia* lethal factor 1 is a cytotoxin which has been reported to interfere with host helicase activity and aid in the process of infection (Cruz-Migoni et al., 2011). Despite *B. mallei* being considered to be a deletion clone of *B. pseudomallei*, the virulence factors from this organism are not as well characterized. *B. mallei* has a T6SS cluster that is homologous to *B. pseudomallei* but does not possess the virulence associated T3SS from *B. pseudomallei* (Memisevic et al., 2013). Further virulence factors (e.g., pili, flagellin) are discussed in more detail later in this review (“Adhesion and Invasion”).

## *Burkholderia* infection of epithelial cells

*Burkholderia* spp. can cause disease by infecting a wide range of human cells (Stevens and Galyov, 2004; Eu et al., 2014). There is substantial literature available on the infection of innate immune cells including macrophages, neutrophils and dendritic cells (Chieng et al., 2012; Horton et al., 2012; Bast et al., 2014). However, during an inhalational infection the pulmonary epithelial cells are one of the first cells to come into contact with bacteria and, along with alveolar macrophages, offer one of the first lines of defense from the organism (Dobos et al., 2000; Sim et al., 2009). The expanding interest in the epithelium as an important player in innate immunity implies that scientific research should consider the role of these cells in combating infection (Eisele and Anderson, 2011). As well as providing a physical barrier the epithelium also plays a role in innate immunity. This includes the direct effects of epithelial-derived antimicrobials such as complement, defensins, lipocalin, lysozyme, nitric oxide, and surfactant (Mason, 2006). The epithelium is also capable of recruiting a variety of immune cells including neutrophils, T- and B-cells and monocytes as well as activating immune cells via secretion of cytokines (Eisele and Anderson, 2011). *Burkholderia* spp. can enter the body through all areas of the respiratory system including the olfactory epithelium leading to colonization and infection of the brain (Owen et al., 2009). This specialized infection of the central nervous system by *Burkholderia* is reviewed elsewhere and is not the focus of this review (Dando et al., 2014). There are a variety of lung epithelial cells now available for the study of infection (Table 1). All of these models have limitations but there is a growing body of work using more complex epithelial models which are more indicative of human infection (Barrila et al., 2010; Duell et al., 2011; David et al., 2014).

### Adhesion and invasion

Adhesion and invasion of host epithelial cells are vital steps during infection appearing to contribute to the overall virulence of *Burkholderia* spp. For example, the *in vitro* infection of alveolar A549 cells by *B. thailandensis* was found to be 10-fold lower when compared to *B. pseudomallei*. This observation was demonstrated to be due to differences between the two species ability to adhere and invade the cells and not as a consequence of intracellular survival (Kespichayawattana et al., 2004; Wongprompitak et al., 2009). Recently, the production of survival protein SurE by *B. pseudomallei* has been hypothesized to be required for invasion of cultured A549 cells suggesting a mechanism for this event (Techawiwattanaboon et al., 2015). Overall, *B. pseudomallei* can infect a range of epithelial cells (Brown et al., 2002), *B. mallei* adheres to but appears not to invade alveolar epithelial cells (Whitlock et al., 2009), whilst *B. cepacia* adherence and invasion is strain dependent (Keig et al., 2001, 2002; Cieri et al., 2002). This pattern also transcends into an *in vivo* setting as clinical isolates of *B. cepacia* show an eight-fold increase in adhesion and invasion of alveolar A549 cells when compared to environmental strains (Tipper et al., 1998). This highlights the importance of this stage of the infection process in determining the overall pathogenicity of the organism. Therefore, by designing treatments that can inhibit the ability of *Burkholderia* spp. to bind to epithelial cells may represent a potential point for therapeutic intervention in the infection cycle. This hypothesis is supported through previously published research that has demonstrated that dextran could inhibit the binding of *B. cepacia* to both A549s and human airway explants (Chiu et al., 2001; Sajjan et al., 2004). In these studies higher weight dextrans were found to have the greatest inhibitory effect on infection by the organism and this highlights the utility of this approach for preventing infection from *B. cepacia*.

There have been numerous studies into potential adherence factors and receptors that *Burkholderia* spp. may require for the initial binding event to occur with host epithelial cells. Two adhesin genes, *boaA* and *boaB*, have been identified via comparative sequence analysis due to their strong similarity to the well-characterized YadA adhesin from *Yersinia enterocolitis*; the first trimeric autotransporter adhesin (TAA) discovered (Casutt-Meyer et al., 2010). *B. mallei* express *boaA* whereas *B. pseudomallei* have been found to express *boaB* as well as *boaA*. It has been hypothesized that *B. pseudomallei* adheres more successfully to alveolar type II cells than *B. mallei* as a consequence of expressing both adherence factors (Lu et al., 2012). The importance of these adhesins has further been demonstrated using knockout mutants where a *B. mallei boaA* knockout (ATCC23344) showed a 50% reduction in adherence to the laryngeal derived HEp2, alveolar A549, and normal human bronchial epithelium cell lines (Balder et al., 2010). In addition by expressing *boaB* in recombinant *Escherichia coli* an increased binding to these epithelial cells was observed (Balder et al., 2010). Other TAAs have been identified in *B. pseudomallei, B. mallei*, and *B. cenocepacia* (Mil-Homens and Fialho, 2012; Lafontaine et al., 2014). For *B. mallei* and *B. cenocepacia*, mutation of the genes (BMA1027 in *B. mallei* and BCAM0219, 0223, and 0224 in *B. cenocepacia*) reduced the ability of the bacteria to bind to laryngeal, bronchial or alveolar human epithelial cells. However, mutation of the TAA in *B. pseudomallei* had no effect (Lafontaine et al., 2014). It may well be that for *B. pseudomallei* the adhesin role is fulfilled by *boaA* and *boaB* whereas for *B. mallei*, which only uses *boaA*, these other TAAs play a more important role in adhesion.

A type IV pilus gene, *pilA*, in *B. pseudomallei*, encodes a protein also involved in adhesion of the bacteria to epithelial cells. The importance of *pilA* has been demonstrated using the knockout *pilA* strain JAB16, with reduced virulence observed in both nematode worms and mice (Essex-Lopresti et al., 2005). Furthermore, the *pilA* mutant displayed reduced adherence to the epithelial cell lines, A549, BEAS2-B, and RPMI-2650, representing the alveoli, bronchi and nasal sections of the respiratory tract. This indicates that *B. pseudomallei* are able to bind to epithelial cells via this receptor-like mechanism ubiquitously in the respiratory tract (Essex-Lopresti et al., 2005). The specific host epithelial factors that allow bacterial adherence are predominantly unknown in *B. pseudomallei* and *B. mallei*. Asialogangliosides GM1 and GM2 are one of the few identified host receptors on pharyngeal epithelial cells facilitating *B. pseudomallei* attachment (Gori et al., 1999). Some Bcc species have also been shown to express proteins that act as adhesins. *B. cepacia* and *B. cenocepacia* express cable pili (Cbl) along with an associated 22 kDa adhesin and their host receptors have been identified. Cbl pili allow the bacteria to attach to host mucins whilst the 22 kDa adhesin allows binding to cytokeratin 13 on the surface of buccal host epithelial cells (Sajjan and Forstner, 1992, 1993). This means the bacteria can still attach even in the absence of mucus. This binding of *B. cepacia* CblA to cytokeratin 13 has been successfully blocked using anti-adhesin antibodies which relieved all pathological effects (Sajjan et al., 2002). The mechanism of these binding events has been studied for *B. cepacia* and *B. cenocepacia* but not for *B. pseudomallei* and *B. mallei* (Sajjan et al., 2002; Urban et al., 2005; Ganesan and Sajjan, 2011). It is likely that *B. pseudomallei* and *B. mallei* may also possess multiple adhesion-type structures in order to ensure that the organisms can mount a successful infection upon host cells under different environmental or physiological conditions. In addition, the functional pathways controlling the expression of proteins involved in adhesion are beginning to be elucidated. For example the knockout of the genes encoding the global regulators BceD and BceF was demonstrated to reduce the adhesion of *B. contaminans* to the CF epithelial cell line CFBE41o- (derived from a CF patient) by four-fold when compared to the wild-type strain (Ferreira et al., 2015). Future work to elucidate the exact mechanism with respect to how the various adhesins interact both with each other and in conjunction with host cell receptors would significantly advance our understanding of the processes involved that underpin the manifestation of disease and will, in turn, potentially facilitate therapeutic intervention at this stage of infection.

The T3SS in pathogenic *Burkholderia* spp. are thought to be important for host cell invasion by the injecting of a range of secretory proteins across the membrane that affect cellular functions. For example, a structural component of the secretion system is encoded for by *bsaQ* and the production of this protein by *B. pseudomallei* can directly affect the invasion of alveolar epithelial cells (Muangsombut et al., 2008). In the absence of *bsaQ B. pseudomallei* invasion dropped by approximately 30% in A549 cells and the organism was unable to secrete T3SS effector proteins (e.g., BopE). The T3SS of *B. pseudomallei* secretes BopE which causes host cell actin rearrangement resulting in membrane ruffling aiding invasion (Stevens et al., 2003). Due to its similarity to the SopE effector protein in *Salmonella* it is believed that BopE functions as a guanine nucleotide exchange factor for the cell cycle regulators Cdc42 and Rac-1 and initiates actin disruption as a consequence (Rudolph et al., 1999). *B. pseudomallei bopE* knock out mutant strains were found to have reduced bacterial load in HeLa cells further highlighting their importance during infection (Stevens et al., 2003). More recently the effects of *B. pseudomallei* Bop family of proteins on epithelial cells have been observed where it was found that knock out *bopC* mutant strains had a reduced ability to invade alveolar A549 cells (Muangman et al., 2011). The role of Bop-induced actin disruption in epithelial cells requires further investigation. Some Bcc species have also been shown to induce actin disruption mirroring the observations found for the highly virulent species of *Burkholderia. Burkholderia cenocepacia* causes actin disruption in primary lung epithelial cells (derived from the bronchi) that had been isolated from CF patients (Sajjan et al., 2006). *Burkholderia multivorans* also causes a similar actin disruption in a differentiated human lung epithelial cell model (Schwab et al., 2003). For the Bcc, it has been found that the rearrangement of actin in epithelial cells is dependent upon microfilaments and microtubules (Taylor et al., 2010).

Other virulence factors thought to be involved in epithelial interactions include flagellin (DeShazer et al., 1997). The mutation of the gene encoding one structural component of the flagellum, *fliC*, led to a decrease in alveolar epithelial cell (A549) invasion (Chuaygud et al., 2008). The complementation of *B. pseudomallei* Δ*fliC* using the gene from both *B. pseudomallei* and *B. thailandensis* shows that both sources of *fliC* restored invasiveness. As such, flagellin does appear to be important in epithelial infection but has led others to comment that its overall role in *in vivo* virulence (i.e., as *B. thailandensis* is avirulent) may be minimal (Allwood et al., 2011). In *B. cepacia*, the mutation of gene encoding the motor-switch component of the flagellum, *fliG*, did not affect adherence but decreased invasion in the same cell type (A549 cells). This suggests that non-functioning flagella can still bind epithelial cells but that motor function is required for infection (Tomich et al., 2002). Further, the mutation of the invasion-related locus, *irl*, led to a significant reduction of *B. pseudomallei* invasion in A549 cells (10% invasion compared to that of the wild-type; Jones et al., 1997). However, the loss of *irl* was also found to have no effect on uptake by phagocytic cell lines or in rodent studies (infant diabetic rat and Syrian hamster challenge models of infection). This suggests that, for reasons that remain unknown, these proteins are specifically required for the infection of epithelial cells.

*Burkholderia* spp. are also able to produce exoproducts that have been shown to affect infectivity in epithelial cells. A bacterial lipase has been identified in Bcc species capable of affecting infection in two bronchiolar epithelial cell models (Mullen et al., 2007). The inhibition of this lipase, with the lipase inhibitor Orlistat, reduced invasion rates in a dose-dependent manner and pre-treatment of the lung epithelial cells with Bcc lipase markedly increased the rate of infection (Mullen et al., 2007). Equivalent research for *B. pseudomallei* and *B. mallei* has as yet not been reported.

### Survival, movement and replication

Once inside host epithelial cells *Burkholderia* species reside in vacuoles where they undergo replication and prevent maturation of lysosomes and are thought to manipulate gene expression in order to slow the maturation/acidification of the endosome (Burns et al., 1996; Sajjan et al., 2006). *B. pseudomallei* and *B. mallei* in particular can circumvent the response from the host by escaping from these matured vacuoles using the T3SS (Stevens et al., 2002; Ulrich and DeShazer, 2004). BopA has been identified as a possible T3SS secreted protein that activates this vacuole escape (Gong et al., 2011). Other gene knockouts for T3SS components (e.g., *bsaZ* and *bsaQ*) have resulted in a reduced intracellular survival, in both epithelial and macrophage cell lines as a consequence of the bacteria being targeted by immune pathways whilst captured within host vacuoles (Pilatz et al., 2006; Gong et al., 2011). This demonstrates the importance of the T3SS during infection of both phagocytic and non-phagocytic cells. After vacuole escape, the bacteria can then reside in the cytoplasm and replicate *en masse* leading to severe pathogenesis in the host (Burns et al., 1996; Ray et al., 2009; French et al., 2011). Bacteria sequestered into autophagosomes undergo host-mediated cell destruction known as autophagy, however during severe cases of disease this is rare. *B. pseudomallei* has also demonstrated an ability to escape from these autophagosomes and hence to avoid immune responses using host actin rearrangement (Allwood et al., 2011). As well as the aforementioned role in bacterial invasion previously discussed, the manipulation of host cell actin is also regarded to be important in bacterial movement to other cells. A large amount of force can be generated by the rearrangement of the actin filaments into polymers which in turn can push the intracellular bacteria into neighboring cells spreading infection (Stevens et al., 2006). BimA has been recognized as a virulence factor produced by *B. pseudomallei* that can instigate this polymerisation event and homologs of this protein have also been identified in both *B. mallei* and *B. thailandensis* (Stevens et al., 2005; Sitthidet et al., 2011).

The mechanism of movement utilized by the highly pathogenic *Burkholderia* spp. in order to move across the epithelial layer has yet to be elucidated. However, some indications of the potential mechanisms that may be used exist from studying Bcc species. For example *B. cenocepacia* and *B. stabilis* have been previously shown to pass through cells by transcytosis and paracytosis, respectively, whilst *B. multivorans* is capable of undergoing both processes to increase intracellular spread (Schwab et al., 2002; Saldías and Valvano, 2009). There is limited supporting data to explain the underlying regulatory network that confers these specific phenotypes to the species of *Burkholderia* identified thus far. Although the role of a small number of bacterial derived proteins have been identified. The translocation of *B. contaminans* across a polarized bronchiolar epithelial cell layer is significantly decreased in the absence of the tyrosine kinase, BceF, and phosphotyrosine phosphatase, BceD, suggesting that these proteins in some way influence translocation In addition, certain Bcc species are found to secrete metalloproteases (e.g., ZmpA) in order to break down the epithelium supporting the spread of infection (Gingues et al., 2005). It is likely that highly virulent strains use a combination of these and other regulatory proteins or dissemination mechanisms and that, collectively, these contribute to the generation of acute infections. The rapidity of spread around of the body for *B. pseudomallei* is highlighted by colonization of other organs being noted within 24 h of infection during murine respiratory models of melioidosis (Lever et al., 2009; Laws et al., 2011).

## The epithelial response to *Burkholderia* infection

A range of immune responses have been demonstrated to be induced following infection of epithelial cells with *Burkholderia* spp. (Figure 1B). Novel treatments that target these host responses (i.e., immune-modulation) offer potential opportunities for reducing bacterial pathogenesis and/or the tissue damage that occurs during an acute infection. The initial binding event of *Burkholderia* spp. to host cell surface receptors triggers the release of pro-inflammatory mediators. These include the activation of NFκβ, Erk, and Akt pathways and induction of a vast number of cytokines such as Interleukins 1β, 6 and 8 (IL-1β, 6 and 8), tumor necrosis factor alpha (TNF-α), monocyte chemotactic protein 1 (MCP-1), and chemokine(C-C motif) ligand 20 (CCL20) from alveolar and bronchiolar epithelial cells (Sim et al., 2009; Lu et al., 2012; Gillette et al., 2013). Interleukin-8, a pro-inflammatory cytokine, is secreted by a variety of epithelial cell types in response to infection with a number of different *Burkholderia* spp. (Palfreyman et al., 1997; Reddi et al., 2003; Kaza et al., 2011; Lu et al., 2012). However, differences have been noted in the cytokines that are produced in response to infection with either *B. pseudomallei* or *B. mallei*. For example *B. pseudomallei* appears to induce a more pronounced pro-inflammatory response (driven by IL-6 and -8) compared to *B. mallei*; with the latter also previously found to produce an IL-10 associated anti-inflammatory response in primary human type 2 pneumocytes (Lu et al., 2012). *B. pseudomallei* and *B. mallei* are capable of causing this IL-8 induction by interacting with cell surface components independently of an internalization event. In the A549 alveolar model of the epithelium this activity is driven by p38 MAP kinase (Utaisincharoen et al., 2005). The toll-like receptors (TLRs) play an important role in *Burkholderia* infection in non-epithelial cells. Briefly, *B. pseudomallei* activates TLR2 and 4 on the cell surface further highlighting that the bacteria do not require internalization in order to be immunomodulatory as it was found that heat killed *B. pseudomallei* also induced this affect (West et al., 2008). In particular Lipid A and LPS are thought to be the key ligands for these TLRs and instigate an inflammatory cascade associated with the NFκβ pathway (Dziarski and Gupta, 2000). TLR2 activation is now thought to cause a deleterious effect on the host in response to *B. pseudomallei* infection by inducing mass inflammation and tissue damage in multiple organs (Wiersinga et al., 2007). More recently, a study has demonstrated that the flagellin of *B. pseudomallei* can also lead to the activation of TLR5 (West et al., 2013). Similarly, *B. thailandensis* has also been found to activate TLR2, 4, and 5 (West et al., 2009). The majority of research into TLR activation and the associated intracellular cascades has been performed using non-epithelial cells. However, limited evidence does exist that also indicates the importance of these receptors in tracheal, bronchiolar, and alveolar epithelial infections (Guillot et al., 2004; Kovach and Standiford, 2011; Wu et al., 2011). For example, flagellin-induced TLR5 activation has been observed during *Pseudomonas aeruginosa* infection of large airway primary epithelial cells (Zhang et al., 2005). Despite these findings, cell-free culture supernatants from Bcc cultures have also been able to stimulate the aforementioned IL-8 immune response (Palfreyman et al., 1997; Fink et al., 2003). This suggests that *Burkholderia* spp. can stimulate an immune response via exoproducts as well as direct binding events.

The release of inflammatory mediators induces cellular recruitment in an effort to clear infection. During *B. mallei* infection the chemoattractant MCP-1 and TLR-activated MyD88 have been identified as particularly important for the recruitment of monocytes and dendritic cells to the site of infection. This influx causes the release of IL-12 which then recruits natural killer (NK) cells to produce interferon gamma (IFN- γ) driven immune cascades. The success of this initial recruitment of monocytes and dendritic cells is thought to be vital in the clearance of infection (Goodyear et al., 2010, 2012). During Bcc infection in CF patients, the influx of neutrophils to the lung epithelium can trigger damaging inflammation (Speert et al., 2002). In mice infected via the pulmonary route with *B. pseudomallei*, extensive neutrophil recruitment is found to occur within the alveolar spaces which is subsequently followed by mononuclear cells during the later stages of infection (West et al., 2012). An influx of immune cells can trigger further signaling pathways associated with immune clearance and inflammation. This self-perpetuating cycle can ultimately lead to the tissue damage and organ failure that is typically found in severe cases of melioidosis and glanders. *Burkholderia* spp. are also capable of modulating other parts of the innate immune system, such as antimicrobial peptides. *Burkholderia* spp. demonstrate strong resistance to these natural defense mechanisms. For example, *B. pseudomallei* is resistant to human alpha-defensin 1 (HNP-1) which is often a key player in bacterial clearance (Goodyear et al., 2010), whilst *B. cepacia* has demonstrated resistance to human beta-defensins (hBDs) 1, 2, and 3 (Speert et al., 2002; Goodyear et al., 2012).

The overall structural epithelial integrity of the lung, specifically through the loss of tight junctions and the modulation of the extracellular matrix, can also be affected following infection by *Burkholderia* spp. The disruption of tight junctions, following the dissociation of the main structural component occludin, has been observed in bronchial epithelial cells following infection with *B. cenocepacia* and *B. contaminans* (Kim et al., 2005; Ferreira et al., 2015). Tight junction complex disruptions have also been found to occur during the infection of A549, 16HBE, and Calu-3 cells with *B. cepacia* (Duff et al., 2006). In addition, the infection of CFBE41o- cells with *B. contaminans* decreases tight junction protein 1, ZO-1, and claudin-1, as well as occludin and indicated that tight junction degradation occurs via a decrease in numerous protein components (Ferreira et al., 2015). Collectively this research highlights how widespread in nature, and therefore the potential importance of, tight junction disruption is in aiding the development of an infectious disease. This will ultimately contribute to the loss of epithelial integrity in the lungs that has now been observed for several species of *Burkholderia* and is hypothesized that this aids in the dissemination of the infection. A continued breakdown of the lung epithelia is also thought to occur following the up-regulation of host matrix metalloproteinase (MMP) expression in response to infection with *Burkholderia* spp. In particular the gelatinases MMP-2 and MMP-9, which breakdown collagen and other extracellular matrix components, have been shown to be up-regulated *in vitro* following infection with *B. cenocepacia* (Wright et al., 2011). Clearly the damage that would be caused to the lung epithelium during infection would allow for extensive bacterial spread into the circulatory systems and therefore contribute to the severity of the disease.

Transcriptomics has been used by several groups to look at the global mRNA changes in response to *Burkholderia* infection in a variety of models and tissues, including blood, liver, and spleen (Pankla et al., 2009; Chin et al., 2010, 2012; Conejero et al., 2015). A recent microarray study of alveolar A549 cells infected with *B. cepacia* identified that the host genes involved in inflammation, apoptosis, and the cell cycle were all down-regulated (Mariappan et al., 2013). By dampening the immune responses and by preventing apoptosis virulent *Burkholderia* strains can create a beneficial environment for replication and survival. Transcriptomics on epithelial cells have yet to be carried out for *B. mallei* but have been used by some groups for *B. pseudomallei* (Wongprompitak et al., 2009). The host response in whole tissue homogenates has also been studied for *B. pseudomallei* (Ulett et al., 2005; Conejero et al., 2015). Unsurprisingly, the response identified numerous immune and inflammatory genes changing in expression (e.g., IL-1, 4, and 15, TNF related genes). Despite the overwhelming immune responses that were induced it has also been possible to elucidate the specific involvement of the epithelia during infection from *in vivo* lung homogenates (David et al., 2012). This suggests that mechanistic studies to derive epithelial host-pathogen interactions would benefit from “*in vivo*-like” co-culture models (Barrila et al., 2010; Duell et al., 2011). Coupling transcriptomics with more complex three dimensional cell models of infection (creating a more “*in-vivo*-like” lung environment) has been utilized successfully to investigate host-pathogen interactions for other bacteria (David et al., 2014). More advanced approaches of this type may aid in the discovery of new targets for therapeutic intervention in the future.

## Concluding remarks

The diseases melioidosis and glanders caused by *B. pseudomallei* and *B. mallei*, respectively, are an enduring issue of international concern. At present the treatments available are limited, protracted and largely ineffective. As a consequence, new approaches are required in order to identify new drugs or drug targets that aid in the clearance of infection. It is clear that the interaction between pathogenic *Burkholderia* spp. and the epithelium is a key determinant in virulence. The interactions between these organisms and the lung epithelium represents an under researched area, which may offer the potential for new therapeutic interventions. Innovations in the field of opportunistic pathogens for the treatment of CF may yield transferable models, drugs or drug targets that could be utilized in order to identify efficacious treatments against infections caused by the highly virulent strains of *B. pseudomallei* and *B. mallei*.

## Author contributions

JD and RB reviewed the data from the literature and organized and wrote the manuscript. GC was involved in writing and editing the final version of the manuscript. All of the authors read and approved the final version of the manuscript.

### Conflict of interest statement

The authors declare that the research was conducted in the absence of any commercial or financial relationships that could be construed as a potential conflict of interest.
